# Functional and in silico evidence for the role of microRNAs 148a-5p and 199a in migration and invasion of papillary thyroid carcinoma cells

**DOI:** 10.1007/s12672-025-03277-6

**Published:** 2025-10-24

**Authors:** Karina Colombera Peres, Mateus Leandro Bezerra, Leonardo Augusto Marson, Mônica Pezenatto Santos, Alexandre Hilário Berenguer Matos, Irena Dus-Ilnicka, Larissa Teodoro Rabi, Alfio José Tincani, Priscila Costa Tincani, Natassia Elena Bufalo, Murilo Vieira Geraldo, Laura Sterian Ward

**Affiliations:** 1 Laboratory of Cancer Molecular Genetics, School of Medical Sciences, University of Campinas (UNICAMP), Campinas, SP Brazil; 2 Max-Planck University Center (UniMAX), Indaiatuba, SP Brazil; 3 Department of Structural and Functional Biology, Institute of Biology, University of Campinas (UNICAMP), Campinas, SP Brazil; 4 Department of Neurology, School of Medical Sciences, University of Campinas (UNICAMP), Campinas, SP Brazil; 5 Oral Pathology Department, Faculty of Dentistry, Wroclaw Medical University, Wroclaw, Poland; 6 Department of Biomedicine, Nossa Senhora Do Patrocínio University Center (CEUNSP), Itu, SP Brazil; 7 Department of Medicine, São Leopoldo Mandic and Research Center, Campinas, SP Brazil

**Keywords:** Metastasis, Lymph node, Thyroid cancer, microRNA

## Abstract

Thyroid cancer remains one of the most prevalent cancers worldwide, with lymph node metastasis (LNM) playing a critical role in determining patient prognosis and treatment. MicroRNAs (miRNAs) are essential post-transcriptional regulators of gene expression and have been implicated in cancer progression, particularly in epithelial-mesenchymal transition (EMT). This study aimed to identify miRNAs involved in the lymph node metastatic process in thyroid cancer and to evaluate their potential as therapeutic targets. RNA-seq datasets from Gene Expression Omnibus (GEO) and The Cancer Genome Atlas (TCGA) were used to identify dysregulated miRNAs in LNM from papillary and medullary thyroid carcinoma (PTC and MTC). The candidate miRNAs were transfected in thyroid carcinoma cell lines, TPC-1 and BCPAP, followed by quantification of target genes and analysis of cell migration and invasion assays in vitro. Three miRNAs *miR-199a-5p*, *miR-199a-3p*, and *miR-148a-5p* were identified as downregulated in thyroid cancer with LNM. Transfection of TPC-1 and BCPAP cells with mimetics reduced cell migration and invasion. Additionally, these miRNAs modulated the expression of EMT-related genes, particularly in BCPAP cells. Our results indicate that miR-199a-3p, miR-199a-5p, and miR-148a-5p are involved in key metastatic processes, suggesting their relevance as potential biomarkers and/or therapeutic targets for thyroid tumors. Further in vivo validation and clinical studies are required to explore their translational applications.

**Supplementary Information:**

The online version contains supplementary material available at 10.1007/s12672-025-03277-6.

## Introduction

Thyroid nodules affect almost a quarter of the global population, regardless of the diagnostic techniques used, and their prevalence has increased in recent years [1]. Although most of these nodules are benign and present no clinical relevance [2], thyroid cancer is an important health issue, accounting for one of the ten most prevalent tumors worldwide [3]. Based on estimates from the American Cancer Society, approximately 44,020 new cases of thyroid cancer are expected in the United States by 2024, with approximately 12, 500 cases occurring in men and 31,520 in women. Additionally, thyroid cancer is projected to cause 2170 deaths, including 990 men and 1,180 women [3]. Over the last 30 years, there has been a notable increase in its prevalence, mainly owing to the use of sensitive diagnostic methods that can identify small tumors that represent a large proportion of currently diagnosed cases. The high economic burden of these tumors, mostly papillary thyroid cancers (PTC) with indolent behavior, has led to the proposal of less invasive measures such as active surveillance [4]. However, a considerable proportion of PTC cases present with regional lymph node metastases (LNM) at diagnosis or during follow-up, which may have prognostic implications, especially in aggressive variants [5] and elderly patients [6]. The lymph node ratio has been proposed as a useful tool for the stratification of patients with PTC and lateral neck metastases [7]. LNM are also important determinants of the prognosis of patients with medullary thyroid cancer (MTC). Sixty percent of patients with MTC < 2 cm already have cervical lymph node involvement at the time of diagnosis, making the surgical procedure the best chance for survival and long-term well-being [8]. In this context, molecular markers of predisposition to LNM could be important in the therapeutic decision-making process for patients with thyroid cancer.

EMT is a fundamental aspect of cancer progression, where tumor cells acquire migratory and invasive properties, facilitating their dissemination throughout the body. This transition is crucial for the development of metastasis. Additionally, cell adhesion plays a vital role in anchoring these migrating cells to new tissues, further promoting metastasis and complicating cancer treatment strategies [9]. Understanding the mechanisms underlying EMT and cell adhesion is essential to develop effective therapeutic interventions against cancer metastasis.

MicroRNAs (miRNAs) have recently emerged as useful biomarkers of cancer [10]. These small non-coding RNAs play a critical role in the regulation of gene expression and are, therefore, involved in the pathogenesis and progression of different types of tumors [10]. Depending on the function of their target genes, miRNAs can act as either oncogenes or tumor suppressors [11]. An increasing number of studies have demonstrated the abnormal expression of miRNA in various types of tumors [12], and clinically available miRNA-based tests have proven useful in the diagnosis and prognosis of patients with thyroid cancer [13]. However, since they act in very complex networks, as one miRNA may regulate many genes as their targets and one gene may be targeted by many miRNAs, their role in the pathogenesis of thyroid tumors is still largely unknown. 

In this study, we used bioinformatics tools to screen PTC and MTC miRNA expression datasets to investigate key deregulated miRNAs that may be essential players in EMT and cell adhesion failure. We assumed that the same important miRNAs may regulate the same pathways in different thyroid carcinoma types [14]. We then validated the in-silico findings, showing the role of the most relevant miRNAs identified in cell migration and invasion assays.

## Materials and methods

### Bioinformatic identification of miRNA expression in thyroid cancer datasets

We used the descriptors for “miRNA AND Thyroid Carcinoma” and “miRNA AND Thyroid Neoplasm” on Gene Expression Omnibus (GEO, https://www.ncbi.nlm.nih.gov/geo/) [15]. GSE104006 [16] and GSE97070 [17] were considered suitable for the analysis in GEO2R (https://www.ncbi.nlm.nih.gov/geo/geo2r/), software provided by the GEO platform for the statistical analysis of gene expression. Additional information is provided in Supplementary Table S1.

Differentially expressed miRNAs from the PTC LNM × non-neoplastic tissue sample group were compared with those from the MTC LNM × non-neoplastic tissue sample group. The exclusion criterion was that these miRNAs should not be downregulated in samples without lymph node metastases. The cutoff point established was an adjusted p-value < 0.05 (by Benjamini and Hochberg’s false findings correction method) and log2 (mean fold change) > 1.

Among the 14 miRNAs commonly dysregulated in both PTC LNM and MTC LNM samples, we selected miR-148a-5p, miR-199a-5p, and miR-199a-3p for further functional validation based on two main criteria: (i) these three miRNAs were consistently downregulated in LNM-positive samples but not deregulated in non-metastatic tumors, suggesting a specific association with metastatic potential rather than with tumorigenesis in general, and (ii) they exhibited statistically significant fold changes and adjusted p-values in both datasets, reinforcing their potential biological relevance across different thyroid carcinoma subtypes. This integrative filtering strategy allowed us to prioritize miRNAs with consistent behavior and functional implications for tumor invasiveness.

In addition, the RNA-seq dataset containing 507 PTC, 8 LN metastasis, and 59 normal samples was downloaded from The Cancer Genome Atlas (TCGA) database (https://portal.gdc.cancer.gov). miRNA expression data are presented as reads per kilobase per million mapped reads (RPKM). We used miRWalk v2.0 [18] to predict miRNA-target interactions using 12 built-in databases for the target genes for each DEM in our study. To increase reliability, only target predictions by at least 7 of the 12 platforms were considered valid.

### Gene expression data

For the gene expression data of PTC with LNM, we searched GEO for the descriptors “papillary thyroid carcinoma [All Fields] AND (“neoplasm metastasis” [MeSH Terms] OR metastasis [All Fields])” and “(“neoplasm metastasis” [MeSH Terms] OR metastasis [All Fields]) AND PTC [All Fields]” which returned 40 results. The exclusion criteria were GSEs unsuitable for GEO2R and lack of PTC LNM and healthy control data. After applying these parameters, we selected three datasets: GSE151179 [19], GSE104006 [16], and GSE60542 [20]. Supplementary Table S2 describes the samples that constituted each type of genomic expression dataset.

Gene expression data of MTC LNM was searched on GEO for “MTC [All Fields] AND (“neoplasm metastasis” [MeSH Terms] OR metastasis [All Fields]),” which returned two results, both of which were excluded from the analysis for not having healthy control data available.

Using GEO2R, we analyzed differentially expressed genes (DEG) between PTC LNM and non-neoplastic tissue samples. We used a cutoff adjusted p-value < 0.05 (by Benjamini & Hochberg’s false findings correction method). Once all the miRNAs identified in our analysis were downregulated, the downregulated genes were excluded from the list.

Finally, to complement the analysis, we used public data downloaded by cBioportal [21] on 500 PTC samples (365 women and 135 men, mean age 47.3 ± 15.8 years old) that presented data for mRNA-seq. These data are presented as transcripts per million (TPM) of mRNA relative to normal expression. We used clinical information available from these samples, such as the American Joint Committee on Cancer TNM staging [22], to conclude gene expression and patient prognosis.

### Cell culture and transfection

Two cell lines with different genetic backgrounds, maintained in our laboratory, were used to evaluate the activity of miR-199a-3p, miR199a-5p, and miR-148a-5p in thyroid cell migration. The human papillary carcinoma-derived strains, TPC-1 and BCPAP, harbor the RET/PTC1 chromosomal rearrangement and BRAFT1799A mutation, respectively [23]. TPC-1 and BCPAP cell lines were maintained in DMEM supplemented with 5% and 10% fetal bovine serum (FBS), respectively, in an incubator under conditions of 95% air and 5% CO2, at 37 °C, in the presence of antibiotic and antifungal solutions. Commercial mimetics of the mirVana^™^ system were used to restore the expression of these miRNAs in the cell lines (miR-148a: Assay ID MC10263, miR-199a-5p: Assay ID MC10893, and miR-199a-3p: Assay ID MC11779, ThermoFisher). TPC-1 and BCPAP lines were plated in triplicate, and after 24 h, the culture medium was replaced with the appropriate medium in the absence of fetal bovine serum. The mimetic was first transfected at four different concentrations (5 nM, 10 nM, 15 nM, and 20 nM) using the cationic lipid Lipofectamine 2000^®^ (Thermo Fisher Scientific), and two concentrations were chosen for further experiments. The group subjected to the transfection procedure without mimetics (mock) was used as the control. The overexpression of each miRNA was confirmed by RT-PCR, as described below.

### Cell migration and invasion

After transfection with the mimetics, 4.5 × 104 cells per well were plated in 12-well plates. Using a sterile tip, we created a vertical lesion in the cell monolayer at the center of each well. The healing process was recorded with a camera attached to the computer soon after the lesion was made (time 0), and then at 16 and 24 h at 40 × magnification. The images obtained from each well were analyzed using ImageJ^®^ software to calculate the distance (in pixels) between the lesions at different times. Each image was measured at three different points using the distance values of pixels collected for statistical applications.

Cell migration analysis was also performed using a modified Boyden chamber containing membrane inserts with 8.0 μm pores. After transfection with mimetics (10 nM and 20 nM), the cells were resuspended in a medium containing FBS and plated in the upper compartment of the chamber. The bottom compartment was filled with a medium containing 10% FBS. After 12 h, the medium was removed, and the chamber was washed with PBS. The cells in the upper compartment were removed and the cells in the lower compartment were fixed, stained with 0.5% Crystal Violet, and photographed using a Nikon Eclipse E600 microscope. Each image was measured at five different points to count the number of cells for statistical analysis.

For invasion analysis, a commercial extracellular matrix (ECM Gel from Engelbreth-Holm-Swarm murine sarcoma-liquid, BioReagent) diluted in DMEM was used. Thirty microliters of the diluted matrix were placed on top of each insert. The inserts were allowed to rest in an oven at 37 °C for two hours to solidify the matrix. After the preparation of the inserts with the matrix, the protocol described above was followed for plating, sample collection, and photography.

### Quantitative reverse-transcribed PCR

MiRNA and target expression were quantified by RT-qPCR. Total RNA was extracted after 72 h of mimetic transfection (3 × 105 cells) based on Chomczynski and Sacchi35. For miRNA, cDNA was synthesized using TaqMan MicroRNA Reverse Transcription Kit (ThermoFisher) using 10 ng of the total RNA. qPCR reactions were performed using the TaqMan Universal Master Mix II, No UNG kit and Taqman MicroRNA Assays for each of the miRNAs (miR-148a: Assay ID 000470, miR-199a-5p: Assay ID 000498, miR-199a-3p: Assay ID 002304 and RNU6B: Assay ID 001093, ThermoFisher). For target genes, 1 µg of total RNA was reverse-transcribed and 5 ng was used for qPCR reaction. The quantification of mRNA target genes was performed using SYBR Green Dye reagent (ThermoFisher) in a final volume of 20 μL. RNU6B and RPL19 were used as endogen controls for miRNA and mRNA quantification, respectively, and the relative quantification was calculated using the 2^-ΔΔCt^ method, as described by Livak and Schmittgen. The reactions were carried out in StepOnePlus equipment (ThermoFisher) and the 7300 SDS Software program was used to analyze the data obtained. *ITGA3* Fw 5′-TATTCCTCCGAACCAGCATC-3′ and Rev 5′-TCCGAGTCAATGTCCACAGA-3′; *NECTIN1* Fw 5′-CAGCCACTGAGTACCACTGG-3′ and Rev 5′-TGCCAGGCTGTAGTTGATGG-3′; *SNN* Fw 5′-TGCCCAACTGTTGCATTCAAG-3′ and Rev 5′-GCTTCTGCAGTGATACGAAGG-3′; *FBXO28* Fw 5′-TCCTCAGCTTTATGTCCTACGA-3′ and Rev 5′-CTGATTCAACATTCTCTGGCAGA-3′; *RPL19* 5′-AACAAGCGGATTCTCATGG -3′ Rev 5′-GCGTGCTTCCTTGGTCTTAG-3′.

### Statistical analyses

All analyses were performed using GraphPad Prism software (version 9.0). Continuous variables were compared using parametric tests (Student’s t-test or ANOVA). Statistical significance was set at p < 0.05.

## Results

### miRNAs expression data

Twenty-five miRNAs were deregulated in PTC LNM samples compared to healthy controls in the GSE104006 dataset (Supplementary Table S3), while 41 miRNAs were deregulated in MTC LNM samples compared to healthy controls in the GSE97070 dataset (Supplementary Table S4). As expected, the most upregulated miRNAs were the previously described *miR-146b-5p*, *miR-222-3p*, *miR-221-3p* in PTC [24] and *miR-375* in MTC [25]. Considering that MTC frequently spreads to local metastases, we searched for common miRNAs between both TC histological subtypes to identify possible LNM-associated miRNAs. This approach resulted in 14 common deregulated miRNAs in both PTC LNM and MTC LNM, with only three not being deregulated in comparison with PTC and MTC samples without LNM. We focused our further analysis on these three miRNAs to explore their possible correlation with LNM metastasis in different thyroid tumors. The fold changes, p-values, and adjusted p-values for *miR-199a-3p*, *miR-199a-5p*, and *miR-148a-5p* are shown in Table 1.

**Table 1:** Deregulated miRNAs in thyroid carcinomas with lymph node metastasis

miRNA	PTC LNM			MTC LNM		
	Log FC	p-value	Adj p-value	Log FC	p-value	Adj p-value
*miR-199a-5p*	− 1.24834	0.000152	0.008906	− 0.971227	0.00195	0.0302
*miR-199a-3p*	− 2.18590	0.000005	0.000785	− 1.238755	0.00155	0.0260
*miR-148a-5p*	− 2.26743	0.000028	0.002838	− 1.693896	0.00555	0.0492

*PTC LNM* papillary thyroid carcinoma with LNM, *MTC LNM* medullary thyroid carcinoma with LNM, *Adj* adjusted

**Fig. 1:**
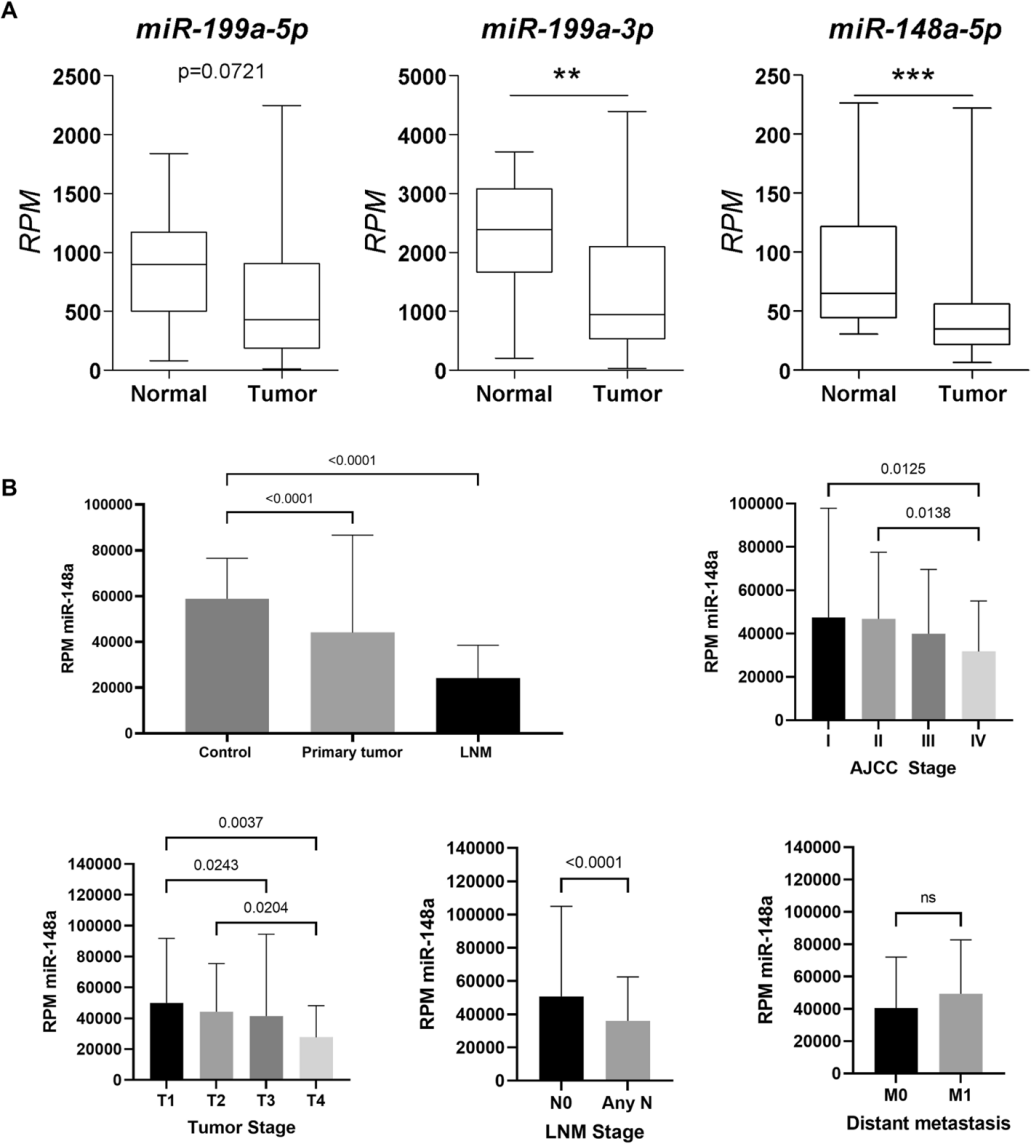
De-regulation of miRNAs in LNM-positive TC. **a** miRNA expression of *miR-199a-5p, miR-199a-3p* and *miR-148a-5p* in the TCGA cohort. **b** *miR-148a* is downregulated in more aggressive papillary thyroid carcinomas. *AJCC* American Joint Cancer Committee; LNM. ****p < 0.0001; ***, ** and *p < 0.05, *NS* not significant

Analysis of RNA-seq data from TCGA confirmed significant downregulation of *miR-148a-5p* and *miR-199a-3p* in tumors compared to that in normal tissues (Fig. 1A). Additionally, reduced *miR-148a* expression was related to the advanced AJCC stage, including the T4 stage and the presence of LNM, but not distant metastasis (Fig. 1B). We were unable to find data for *miR-199a-3p* and *miR-199a-5p* in the TCGA dataset.

### Gene expression data

Using miRWalk v2.0, we searched for potential target genes of the three differentially expressed miRNAs. Nine common targets were identified: *SNN*, *PVRL1* (also called *NECTIN1*), *FBXO28*, *ITGA3*, *FXR1*, *RC3H1*, *ANKRD52*, *VANGL1*, and *LCOR*. Accordingly, examination of transcriptomic data from thyroid tumors revealed the upregulation of *ITGA3*, *NECTIN1*, *SNN*, and *FBXO28* genes in LNM thyroid carcinomas when compared to normal tissues (Table 2), highlighting them as possible target genes of the selected miRNAs.

**Table 2:** Log_2_ foldchange, p-value, and adjusted p-value data of predicted genes in lymph node metastasis thyroid carcinoma compared with normal tissue

Gene	Log2 foldchange	p-value	Adj p-value
*SNN*^*a*^	0.312786	0.000645	0.004580
*PVRL1*^*b c*^	0.898478	0.000008	0.000290
	1.10	0.000513	0.016069
*FBXO28*^*a*^	0.237112	0.003540	0.025300
	0.290343	0.000123	0.001160
*ITGA3*^*c*^	1.31	0.002300	0.038743
*FXR1*^*b*^	− 0.509844	0.000451	0.005630
*RC3H1*^*a*^	− 0.348581	0.000022	0.000281
*VANGL1*^*a*^	− 0.821988	0.000004	0.000168

Adj adjusted, ^a^ GSE60542, ^b^ GSE151179, and ^c^ GSE104006

**Fig. 2:**
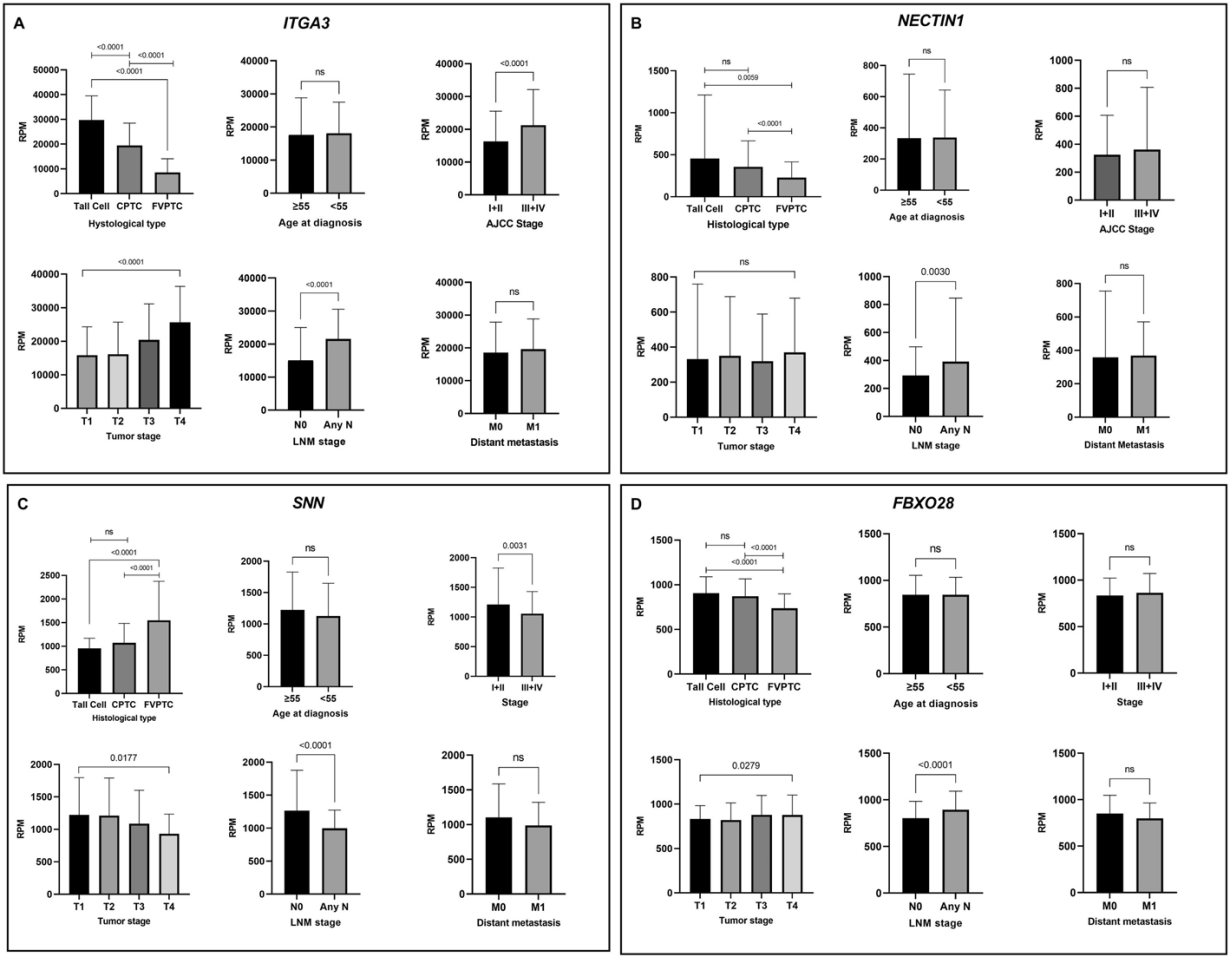
Expression of the predicted *ITGA3* (**a**), *NECTIN1* (**b**), *SNN* (**c**), and *FBXO28* (**d**) genes in papillary thyroid carcinomas and their correlation with poor prognosis. *CPTC* classic papillary thyroid carcinoma, *FVPTC* follicular variant of PTC, *AJCC* American Joint Cancer Committee, *LNM* lymph node metastasis; ****p < 0.0001, **p < 0.05, *NS* not significant

TCGA data showed higher expression of the *ITGA3* gene in tall cell PTC as compared to CPTC and FVPTC (all p < 0.0001, Fig. 2A). Similarly, this was observed for *NECTIN1* (Fig. 2B) and *FBXO28* (Fig. 2D), but not for *SNN* (Fig. 2C). Higher expression of *ITGA3* was also correlated with advanced AJCC stages (III-IV versus I-II, p < 0.0001); hence, we found overexpression in the T4a stage (p < 0.0001) and at any N stage (N1, N1a, N1b versus N0, p < 0.0001). LNM tumors also showed significantly higher expression of *NECTIN1* (p = 0.0030) and *FBXO28* (p < 0.0001) genes, and *SNN* showed higher expression in tumors without LNM than in tumors N1, N1a, and N1b (p < 0.0001). In fact, *SNN* showed an inverse pattern compared to other evaluated genes: expression of *SNN* was higher in FVPTC and in tumors with AJCC stage I and II, T1-T2, and N0 (Fig. 2C). None of the analyzed genes were related to distant metastasis, possibly because a small number of these samples had distant metastasis (M1 = 9) and most of them did not have distant metastasis evaluated (Mx = 212).

**Fig. 3:**
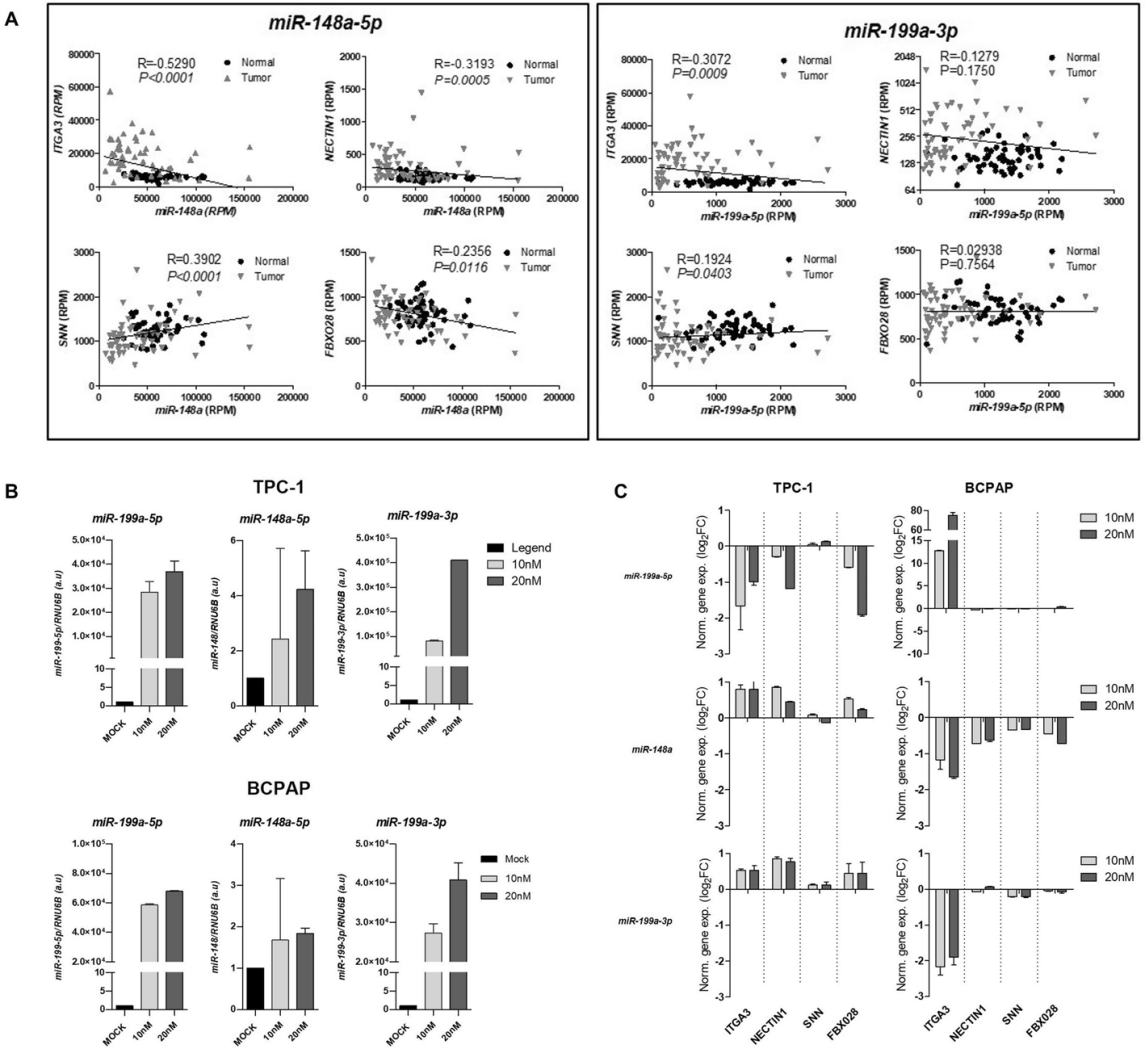
Influence of *miR148a-5p*, *miR-199a-3p,* and *miR-199a-5p* on the expression of target genes *ITGA3, NECTIN1, SNN*, and *FBXO28* in papillary thyroid cancer cell lines. **a** Correlation between *miR-148a-5p*, *miR-199a-3p*, and their targets in human PTC samples according to TCGA gene expression data. **b** Expression of target miRNAs in thyroid cells following transfection with mimics, evaluated by real-time PCR. Results represent the mean ± standard deviation of independent experiments. **c** Predicted expression of miRNAs *miR148a-5p*, *miR-199a-3p*, and *miR-199a-5p* and their targets in papillary RET/PTC (TPC-1) and BRAF mutation (BCPAP) thyroid neoplastic cell lines

Data downloaded from TCGA were used to assess the correlation between the levels of both miRNAs and the identified targets in 57 PTCs and paired non-tumor tissues. As shown in Fig. 3A, linear regression analysis revealed a significant negative correlation between both *miR-148a-5p* (R = − 0.5290, p < 0.0001) and *miR-199a-3p* (R = − 0.3072, p = 0.0009) and *ITGA3*. A significant negative correlation was also observed between *miR-148a-5p* and *NECTIN1* (R = − 0.3193, p = 0.0005) and *FBXO28* (R = − 0.2356, p = 0.0116).

We next evaluated the impact of each miRNA restoration on the expression of the candidate target genes *ITGA3*, *NECTIN1*, *SNN*, and *FBOX28* using quantitative real-time PCR and cDNA from the cell cultures transfected with mimetics (Fig. 3B). Notably, the relative expression of the target genes varied upon the transfection of each miRNA across the cell lines. In BCPAP cells, the transfection of *miR-148a-5p* led to marked decreased levels of the upregulation of *ITGA3*, *NECTIN1*, and *FBXO28* whereas these genes were upregulated in TPC-1 cells. Restoration of *miR-199a-3p* induced similar patterns, with particularly strong downregulation of *ITGA3* in BCPAP cells. In contrast, transfection with *miR-199a-5p* led to *ITGA3* downregulation in TPC-1 cells and its upregulation in BCPAP cells. These results suggest that miRNA’s biological role may be influenced by the genetic background of each cell line.

### Migration and invasion data

**Fig. 4:**
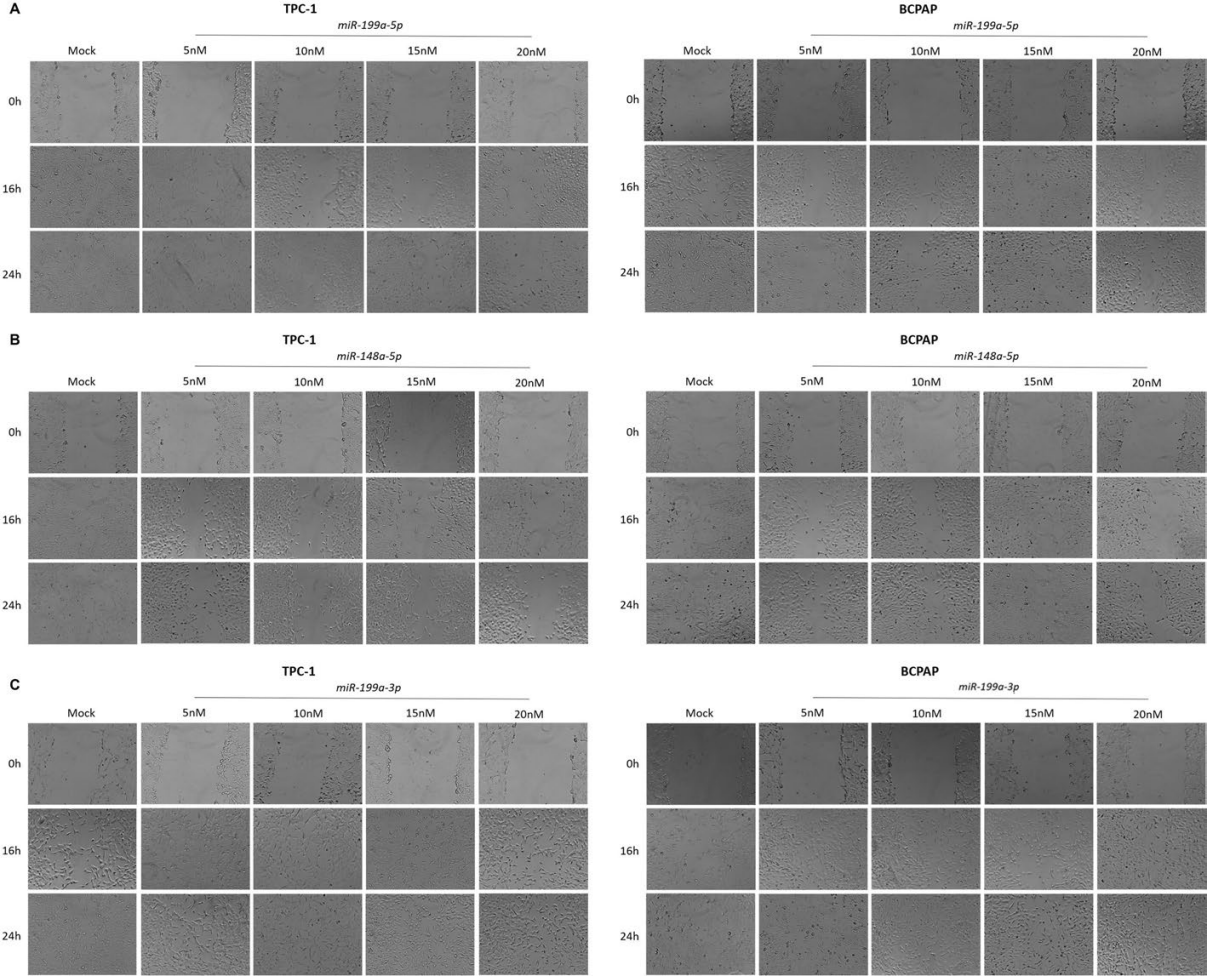
*miR-199a-5p*, *miR-148a-5p*, and *miR-199a-3p* and the cell migration in thyroid cancer cells. After transfection of 4.5 × 104 with each mimetic, the cells were allowed to cover the plate, and a wound was performed with a pipette tip. The cells were monitored for 24 h, and the area of the scratch was quantified

As the modulation of cell adhesion molecules is a crucial step in migration and invasion during metastatic dissemination, two different assays were employed to investigate the roles of the three miRNAs (*miR-199a-3p*, *miR-199a-5p*, and *miR-148a-5p*) in the migration and invasion of thyroid cancer cells. In the wound healing assay, we found that restoring *miR-148a-5p* and *miR-199a-5p* expression in both TPC-1 and BCPAP cells significantly reduced the number of cells migrating to close the wound compared to the mock control (Fig. 4). Interestingly, this effect was not observed with *miR-199a-3p*.

**Fig. 5:**
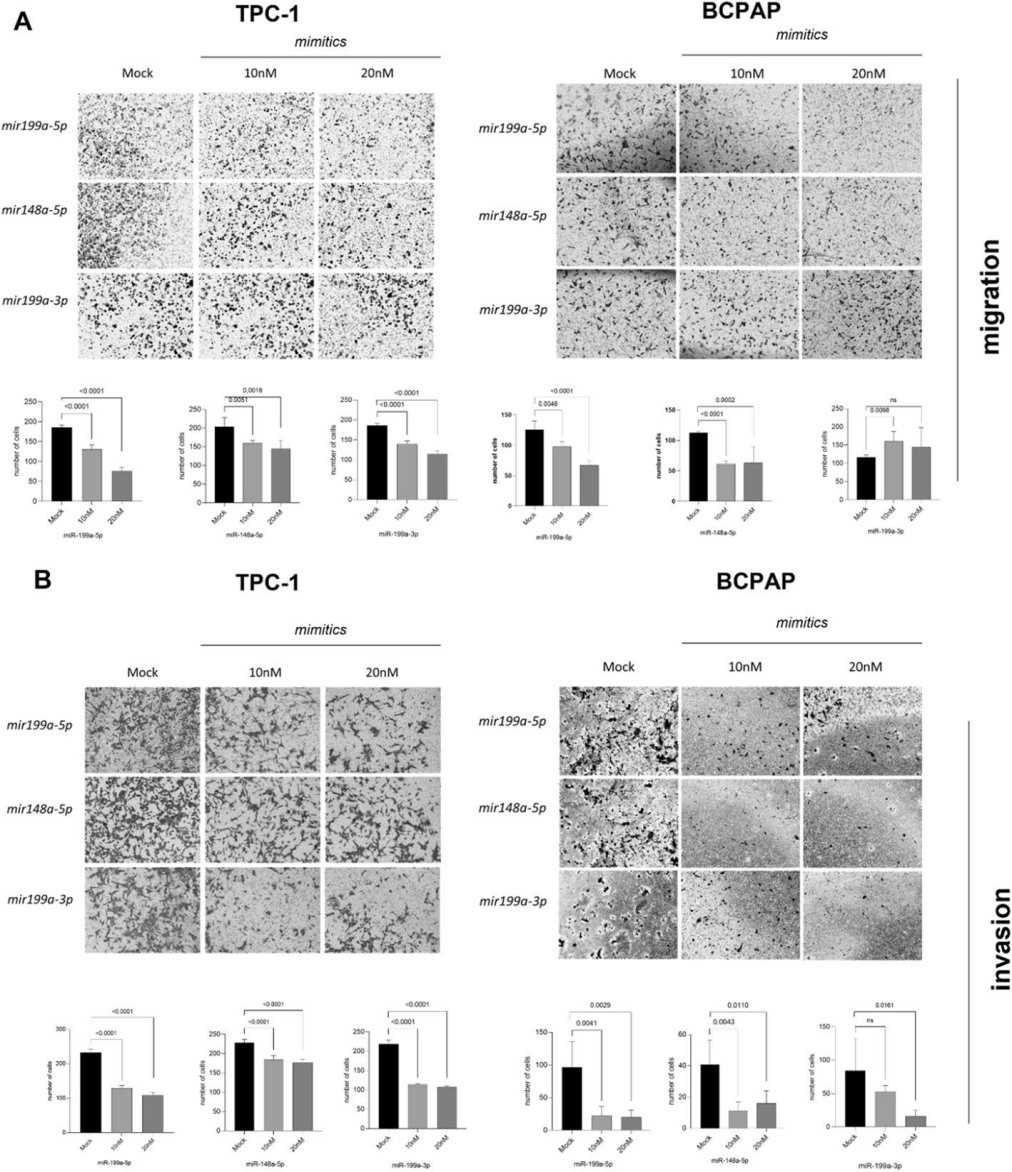
*miR-199a-5p*, *miR-148a-5p*, and *miR-199a-3p* and the cell migration and invasion in thyroid cancer cells. Transwell migration (**a**) and invasion (**b**) assays were performed in TPC-1 and BCPAP cells. The results showed a decrease in both migration and invasion in both cells for *miR-199a-5p* and *miR148a-5p*. The average number of migrating and invading cells is presented as a bar chart with mean ± standard deviation

In the Transwell migration assay (Fig. 5A), the images showed a decreased density of migratory cells in both TPC-1 and BCPAP cell lines after restoring *miR-148a-5p* and *miR-199a-5p* levels. Interestingly, transfection with the *miR-199a-3p* mimic had opposite effects on migration between the two cell lines; migration was reduced in TPC-1 cells but increased in BCPAP cells (Fig. 5A). Regarding the role of these miRNAs in the invasion process (Fig. 5B), transfection with *miR-148a* and *miR-199a-5p* mimetics led to a reduction in the invasive capacity of neoplastic TPC-1 and BCPAP cells. Additionally, restoration of *miR-199a-3p* expression reduced the invasive potential of TPC-1 cells, but in BCPAP cells, this effect was observed only at a higher concentration (20 nM).

**Fig. 6:**
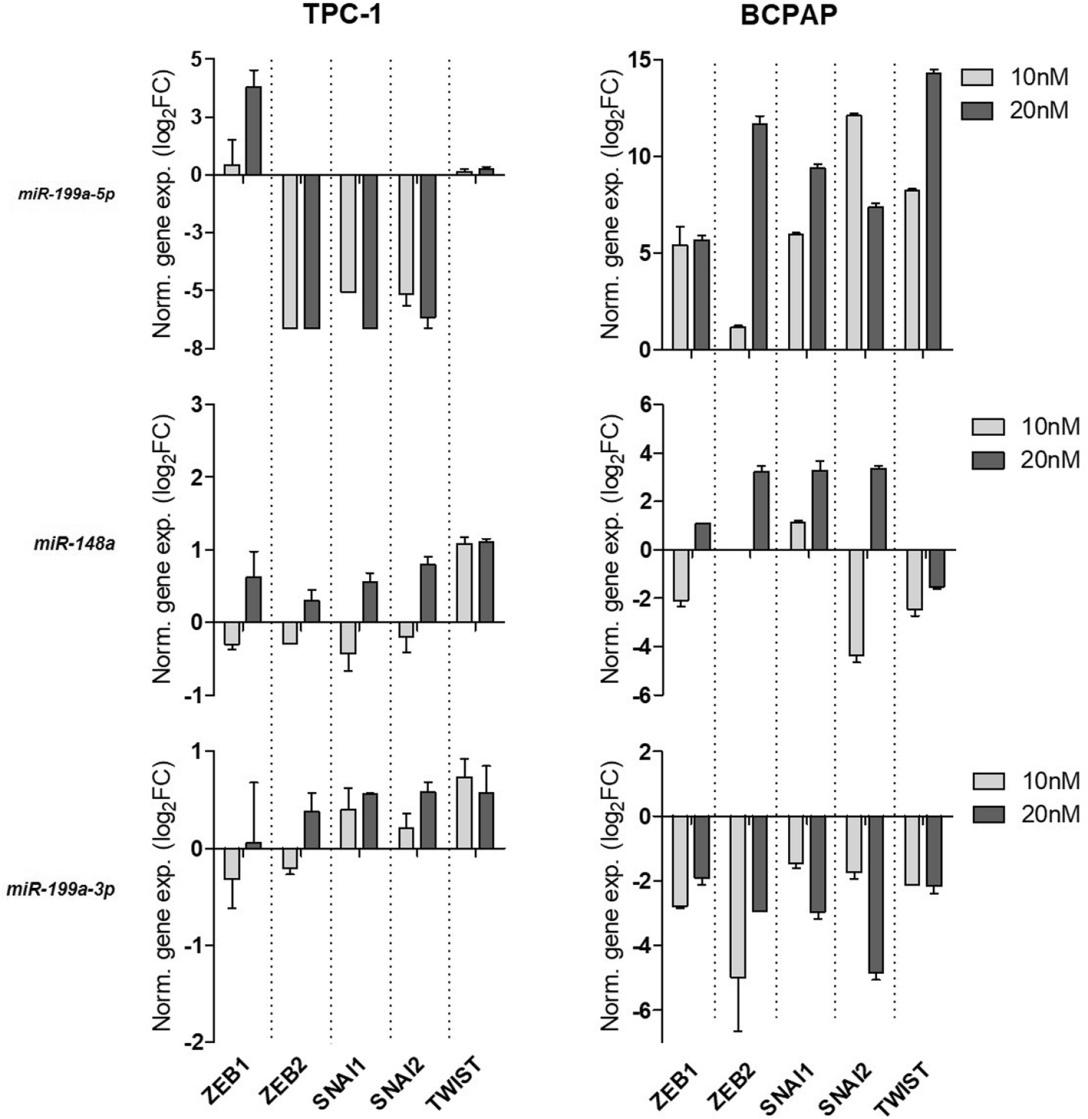
Effect of *miR-148a-5p* and *miR-199a-5p/3p* mimetics on EMT-Related Gene Expression in vitro. TPC1 and BCPAP Cell Lines were transfected with commercial mimetics for each miRNA. After 72 h, the total RNA was extracted and reverse transcribed. *RPL19* was used as endogenous control

To evaluate the impact of restoration of these miRNAs on EMT, we quantified the expression of the key players *ZEB1* and 2, *SNAI1* and 2 and *TWIST1*. The transfection of *miR-199a-3p* mimic in BCPAP showed a significant reduction in EMT-related gene expression. In contrast, this effect was not observed in TPC1 cells. Transfection of TPC1 and BCPAP cells with the *miR-148a-5p* mimic resulted in contrasting gene expression changes depending on the concentration, without clearly indicating a role for this miRNA in regulating the genes involved in EMT. Importantly, TPC1 cells transfected with 20 nM nM *miR-199a-5p* mimic exhibited a marked reduction in the expression of the EMT-related genes, despite overexpression of *ZEB1*. Meanwhile, in BCPAP cells, transfection with 10 nM and, more notably, 20 nM of the *miR-199a-5p* mimic led to the overexpression of all EMT-related genes (Fig. 6).

## Discussion

Although the impact of LNM on PTC prognosis remains a matter of debate, its presence significantly affects patient management [26]. As the same miRNAs may regulate the same pathways in different carcinomas, we employed robust bioinformatics tools to search for miRNAs involved in the metastatic process of these two types of thyroid cancer. We identified three miRNAs, and four target genes related to LNM in both PTC and MTC, suggesting that they may have central roles in cell adhesion, migration, and invasion, thus facilitating tumor extension and metastasis. Using two different papillary carcinoma cell lines, we suggested the participation of *miR-199a-5p*, *miR199a-3p,* and *miR-148a-5p* in neoplastic thyroid cell migration and invasion.

Both *miR-199a-3p* and *-5p* isoforms have been previously linked to worse prognosis of thyroid tumors [27–29]. *miR-199a-3p* appears to play a tumor suppressor role in PTC and is downregulated in both in vitro and in vivo studies [28, 29]. When restored in cell lines, *miR-199a-3p* reduces MET and mTOR protein levels, impairing the migration and proliferation of cancer cells [30]. In our study, transfection of *miR-199a-3p* mimetics significantly reduced the expression of epithelial-mesenchymal transition (EMT)-related genes in BCPAP cells only, suggesting a context-specific role in suppressing EMT.

Similarly, *miR-199a-5p* was found to be downregulated in PTC [31] and anaplastic thyroid cancer (ATC) and is strongly associated with cell migration, invasion, and EMT [32]. *SNAI1* was previously identified as a possible target gene of *miR-199a-5p*, reinforcing its role in metastasis. *SNAI1* is a major mediator of invasion and metastasis in different types of cancer because it is responsible for E-cadherin suppression and EMT induction [33]. Our results showed that *miR-199a-5p* has a differential modulatory effect on TPC1 and BCPAP cell lines. In TPC1, transfection with 20 nM of the *miR-199a-5p* mimic led to the overexpression of *ZEB1* and *TWIST*, which are known to promote EMT by inhibiting epithelial genes and increasing the expression of mesenchymal markers, and a significant reduction in other EMT-related genes [34, 35]. Interestingly, we observed an increase in the expression of EMT-related genes after the restoration of *miR-199a-5p* expression in BCPAP cells, whereas our functional analysis showed a marked reduction in cell migration and invasion.

These findings suggest the involvement of additional molecular mechanisms in the regulation of EMT in these cells. Notably, BCPAP harbors the BRAF V600E mutation, which constitutively activates the MAPK pathway and modulates EMT via TGF-β signaling and miRNA deregulation [36]. This crosstalk can lead to a dual role of TGF-β in promoting proliferation arrest in normal cells and EMT in cancer cells, depending on the genetic context. Furthermore, TGF-β signaling has been implicated in the post-transcriptional regulation of miRNAs, including the maturation of miR-199a, further supporting the possibility of a feedback loop in EMT modulation [37].

This phenotype may reflect partial epithelial-mesenchymal transition (p-EMT), a hybrid state in which cells co-express epithelial and mesenchymal markers, limiting migratory potential despite changes in gene expression [38]. Alternatively, it could arise from non-EMT-dependent migration inhibitory mechanisms, such as direct regulation of cell adhesion molecules, including integrins [39]. These possibilities may resolve the apparent paradox observed in BCPAP cells, where *miR-199a-5p* upregulates EMT-related genes (e.g., *ZEB1* and *SNAI1*) without enhancing invasive capacity, suggesting that a non-invasive p-EMT state is potentially regulated by alternative pathways involving adhesion molecules. Notably, *miR-199a-5p* downregulates *ITGA3*, which encodes a key integrin implicated in proliferation, migration, and EMT, via the MAPK and TGF-β pathways [40–42]. Supporting this, our TCGA analysis revealed elevated *ITGA3* expression in aggressive PTC subtypes, including the tall cell variant, tumors with LNM, T4a stage, and advanced clinical stages, corroborating prior findings [43]. Intriguingly, while *miR-199a-5p* reduced *ITGA3* expression in TPC-1 cells, this effect was absent in BCPAP cells, which harbor the *BRAF* V600E mutation—a known activator of the MAPK pathway that regulates integrin expression and extracellular matrix remodeling [44, 45]. Furthermore, other adhesion molecules (e.g., *ICAM-1*, *SELL*, *ITGAL*) have been associated with tumor aggressiveness in thyroid cancer [46]. Collectively, these findings suggest that *miR-199a-5p* modulates invasion through adhesion-related pathways, offering a plausible explanation for the discordance between EMT gene expression and functional behavior in BCPAP cells.

In thyroid tumors, *miR-148a* was found to be downregulated in cancer stem cell (CSC) culture from a patient with anaplastic thyroid cancer (ATC). Overexpression of *miR-148a* in ATC-CSCs induces cell cycle arrest and results in the loss of stem cell characteristics [47]. In another study on ATC, the authors discovered that *miR-148a* could potentially regulate PD-L1 transcription [48]. PD-L1 plays a crucial role in immune evasion and has been shown to significantly suppress the immune response in ATC. Its high expression in ATC tumor tissues is a strong predictor of both progression-free and overall survival in patients [49]. *miR-148a* regulation was also previously investigated by Han et al. [50], who demonstrated its lower expression in thyroid tumor tissues than in adjacent normal tissues, as reported in the TCGA database. Their study also showed that *miR-148a* suppressed proliferation, migration, and invasiveness in two different papillary thyroid cancer cell lines (TPC-1 and K1). Furthermore, the authors suggested that *miR-148a* regulates *CDK8* gene expression, which was confirmed by further experiments demonstrating downregulated *CDK8* expression in TPC-1 and K1 cells transfected with *miR-148a* mimetics, supporting the role of miR-148a in suppressing PTC growth and metastasis by targeting CDK8 [50]. Using TCGA data, we identified a significant association between the downregulation of *miR-148a* and tumors with advanced AJCC stages, such as T4, as well as the presence of LNM. Interestingly, this association was not observed in patients with distant metastases. In line with these findings, although *miR-148a-5p* showed changes in expression in both TPC1 and BCPAP cell lines during our invasion assays, it did not play a clear role in regulating EMT-related genes. This suggests that while *miR-148a* may contribute to certain aspects of tumor progression, such as local invasion and LNM, its role in regulating EMT and promoting distant metastasis may be limited or influenced by other regulatory factors.

According to our functional annotation results, *PVRL1*, also known as *NECTIN1*, is involved in both cell adhesion and EMT. *PVRL1/NECTIN1* gene encodes one of the four members of the cell-adhesion molecules within the immunoglobulin superfamily (IgSF) and plays a key role in both adherent and tight junctions [51]. Although the restoration of the investigated miRNAs did not significantly alter *NECTIN1* expression in TPC and BCPAP cells in our study, high *NECTIN1* expression is associated with poor outcomes in patients with breast cancer [51]. *NECTIN1* is an entry receptor whose expression is increased in the presence of HSV-1 in thyroid tumors and is further increased in papillary thyroid cancer [52]. Our group has previously suggested a relationship between herpes virus infection and the development of malignant thyroid nodules [53]. Additionally, *NECTIN1* has been established as a biomarker of thyroid cancer sensitivity to herpes oncolytic therapy, guiding the selection of treatment programs [54].

Analysis of publicly available data from TCGA database revealed higher *SNN* expression in thyroid tumors without lymph node metastasis, suggesting a potential compensatory role or involvement in growth regulation. Although *SNN* has been associated with TNF-α signaling and cell cycle control [55], its increased expression in non-metastatic tumors may reflect a cellular attempt to restrain early tumor progression. These findings point to a possible metastasis-suppressive function of *SNN*, but further functional studies are needed to clarify its biological role in thyroid carcinomas.

Our results highlight the essential roles of *miR-199a-3p*, *miR-199a-5p*, and *miR-148a-5p* in the regulation of lymph node metastasis in thyroid cancer, particularly through their involvement in epithelial-mesenchymal transition and cell adhesion pathways. The identification of molecular targets such as *ITGA3*, *NECTIN1*, and *FBXO28* provides new insights into the mechanisms driving tumor progression and metastatic dissemination. Functional assays demonstrated that restoring the expression of these miRNAs reduced tumor cell migration and invasion, supporting their potential tumor-suppressive roles. However, the observed differences in miRNA effects between TPC-1 and BCPAP cell lines suggest that their biological function may be context-dependent, influenced by genetic backgrounds such as the presence of the BRAF mutation. This underscores the importance of further investigations to elucidate the precise molecular mechanisms underlying these differential responses.

While our findings suggest that these miRNAs may serve as molecular biomarkers or therapeutic targets, additional validation using in vivo models and patient-derived samples is necessary. Furthermore, future studies should explore potential side effects associated with miRNA modulation and investigate whether these findings extend to other thyroid carcinoma subtypes or cancers of different origins.

## Supplementary Information

Supplementary material 1.

## Data Availability

These data were derived from the following resources available in the public domain: [Gene Expression Omnibus] [https://www.ncbi.nlm.nih.gov/geo/)] [GSE104006, GSE97070, GSE151179 and GSE60542] and [cBioPortal] [https://www.cbioportal.org/]. Also, all data is provided within the manuscript or supplementary information files.
