# Supplementary material for: Functional and in silico evidence for the role of microRNAs 148a-5p and 199a in migration and invasion of papillary thyroid carcinoma cells
Source: Discov Oncol. 2025 Oct 24;16:1962. doi: 10.1007/s12672-025-03277-6 (PMC12552207; doi:10.1007/s12672-025-03277-6)
Supplement: Supplementary file 1 — Supplementary material 1. [file 12672_2025_3277_MOESM1_ESM.docx]

**Role of miR-148a-5p and miR-199a in Cell Migration and Invasion in Papillary Thyroid Carcinoma: Insights from In Silico and Functional Analyses**

**Peres, KC et al.**

**Supplementary material**

**S1.**  Number of samples by histological type in miRNA expression data.

| **Study** | **PTC** | **PTC LMN** | **Normal1** | **PTC+PDTC** | **PDTC** | **MTC LMN** | **MTC** | **Normal2** |
| --- | --- | --- | --- | --- | --- | --- | --- | --- |
| **GSE104006** | 20 | 2 | 6 | 2 | 4 |  |  |  |
| **GSE97070** |  |  |  |  |  | 9 | 8 | 3 |

PTC papillary thyroid carcinoma, PTC LNM papillary thyroid carcinoma with lymph node metastasis, MTC medullary thyroid carcinoma, MTC LNM medullary thyroid carcinoma with lymph node metastasis, Normal1 normal thyroid tissue, Normal2 normal parathyroid tissue, PTC+PDTC sample with papillary thyroid carcinoma and poorly differentiated thyroid carcinoma, PDTC poorly differentiated thyroid carcinoma.

**S2.** Number of samples by histological type in gene expression data.

| **Study** | **PTC** | **PTC LMN** | **Normal** | **NLN** | **PM** | **PTC+PDTC** | **PDTC** |
| --- | --- | --- | --- | --- | --- | --- | --- |
| **GSE151179** | 17 | 17 | 13 |  |  |  |  |
| **GSE60542** | 33 | 23 | 30 | 4 | 1 |  |  |
| **GSE104006** | 20 | 2 | 6 |  |  | 2 | 4 |

PTC papillary thyroid carcinoma, PTC LNM papillary thyroid carcinoma with lymph node metastasis, Normal normal thyroid tissue, PTC+PDTC sample with papillary thyroid carcinoma and poorly differentiated thyroid carcinoma, PDTC poorly differentiated thyroid carcinoma, NLN normal lymph node, PM pleural metastasis.

**S3.** Deregulated miRNAs in PTC LNM in comparison to their normal controls (GSE104006):

| miRNA name | $\mathbf{Log}_{\mathbf{2}}\mathbf{(Fold Change)}$ | P-value | Adjusted p-value |
| --- | --- | --- | --- |
| hsa-miR-199b-5p | -3,76 | 1,22E-07 | 8,23E-05 |
| hsa-miR-204-5p | -3,31 | 1,50E-05 | 1,78E-03 |
| hsa-miR-148a-5p | -2,27 | 2,76E-05 | 2,84E-03 |
| hsa-miR-365a-3p | -2,21 | 2,34E-06 | 6,00E-04 |
| hsa-miR-152 | -2,21 | 1,17E-05 | 1,50E-03 |
| hsa-miR-199a-3p | -2,19 | 4,59E-06 | 7,85E-04 |
| hsa-miR-193b-3p | -1,73 | 1,19E-04 | 8,35E-03 |
| hsa-miR-30c-5p | -1,57 | 2,04E-05 | 2,25E-03 |
| hsa-miR-660-5p | -1,49 | 9,11E-04 | 3,41E-02 |
| hsa-miR-100-5p | -1,45 | 4,71E-04 | 2,27E-02 |
| hsa-miR-99a-5p | -1,40 | 2,06E-04 | 1,14E-02 |
| hsa-miR-193a-3p | -1,38 | 9,50E-04 | 3,41E-02 |
| hsa-miR-95 | -1,36 | 8,94E-04 | 3,41E-02 |
| hsa-miR-199a-5p | -1,25 | 1,52E-04 | 8,91E-03 |
| hsa-miR-192-5p | -1,17 | 4,48E-04 | 2,22E-02 |
| hsa-miR-195-5p | -1,16 | 4,49E-05 | 4,33E-03 |
| hsa-let-7g-5p | -1,13 | 7,03E-05 | 5,72E-03 |
| hsa-miR-28-5p | -1,11 | 1,14E-04 | 8,35E-03 |
| hsa-miR-30a-3p | -1,10 | 1,41E-04 | 8,91E-03 |
| hsa-miR-26b-5p | -1,10 | 1,18E-03 | 4,05E-02 |
| hsa-miR-218-5p | -1,09 | 8,73E-04 | 3,41E-02 |
| hsa-miR-30e-3p | -1,08 | 1,55E-04 | 8,91E-03 |
| hsa-miR-362-3p | -1,06 | 8,17E-04 | 3,40E-02 |
| hsa-miR-374a-5p | -1,06 | 9,50E-04 | 3,41E-02 |
| hsa-miR-215 | -0,92 | 8,06E-04 | 3,40E-02 |
| hsa-miR-574-3p | -0,91 | 1,53E-03 | 4,90E-02 |
| hsa-miR-20b-5p | -0,91 | 1,56E-04 | 8,91E-03 |
| hsa-let-7a-5p | -0,89 | 3,40E-04 | 1,75E-02 |
| hsa-miR-20a-3p | -0,55 | 5,40E-04 | 2,52E-02 |
| hsa-miR-4450 | 0,25 | 1,31E-03 | 4,39E-02 |
| hsa-miR-222-5p | 0,26 | 8,40E-04 | 3,40E-02 |
| hsa-miR-548x-3p | 0,29 | 5,57E-04 | 2,52E-02 |
| hsa-miR-30d-3p | 0,46 | 6,69E-04 | 2,94E-02 |
| hsa-miR-196a-5p | 0,47 | 7,06E-05 | 5,72E-03 |
| hsa-miR-3198 | 0,56 | 1,48E-03 | 4,87E-02 |
| hsa-miR-15a-5p | 0,89 | 3,26E-07 | 1,26E-04 |
| hsa-miR-181d | 0,97 | 1,11E-03 | 3,90E-02 |
| hsa-miR-146b-3p | 1,07 | 3,64E-06 | 7,02E-04 |
| hsa-miR-181a-2-3p | 1,16 | 8,25E-05 | 6,36E-03 |
| hsa-miR-21-5p | 1,47 | 9,39E-06 | 1,45E-03 |
| hsa-miR-181b-5p | 1,51 | 2,99E-04 | 1,59E-02 |
| hsa-miR-221-5p | 1,76 | 1,11E-06 | 3,42E-04 |
| hsa-miR-181a-5p | 2,16 | 1,07E-05 | 1,50E-03 |
| hsa-miR-31-5p | 2,46 | 1,29E-04 | 8,62E-03 |
| hsa-miR-551b-3p | 2,83 | 6,88E-05 | 5,72E-03 |
| hsa-miR-221-3p | 3,96 | 1,60E-07 | 8,23E-05 |
| hsa-miR-222-3p | 5,44 | 1,04E-08 | 1,60E-05 |
| hsa-miR-146b-5p | 6,05 | 3,18E-06 | 7,00E-04 |

**S4.** miRNAs deregulated in MTC LM in comparison to their normal controls (GSE97070):

| miRNA name | $\mathbf{Log}_{\mathbf{2}}\mathbf{(Fold Change)}$ | P-value | Adjusted p-value |
| --- | --- | --- | --- |
| hsa-miR-30a-5p | -3,67 | 1,06E-10 | 7,16E-08 |
| hsa-miR-130a-3p | -2,73 | 2,02E-07 | 5,11E-05 |
| hsa-let-7i-5p | -2,46 | 5,10E-05 | 4,31E-03 |
| hsa-miR-100-5p | -2,25 | 1,02E-07 | 2,96E-05 |
| hsa-miR-126-3p | -2,25 | 8,08E-05 | 5,12E-03 |
| hsa-miR-138-5p | -1,97 | 3,70E-07 | 8,34E-05 |
| hsa-miR-30c-5p | -1,96 | 4,81E-05 | 4,24E-03 |
| hsa-miR-30a-3p | -1,73 | 2,92E-11 | 2,96E-08 |
| hsa-miR-148a-5p | -1,69 | 5,55E-03 | 4,92E-02 |
| hsa-miR-135a-5p | -1,59 | 2,57E-04 | 1,07E-02 |
| hsa-miR-30e-5p | -1,49 | 8,06E-05 | 5,12E-03 |
| hsa-miR-145-5p | -1,34 | 5,54E-05 | 4,32E-03 |
| hsa-let-7b-5p | -1,29 | 5,39E-03 | 4,83E-02 |
| hsa-miR-204-5p | -1,27 | 1,11E-11 | 2,24E-08 |
| hsa-miR-199a-3p | -1,24 | 1,55E-03 | 2,60E-02 |
| hsa-miR-424-5p | -1,15 | 4,59E-03 | 4,45E-02 |
| hsa-miR-630 | -1,12 | 7,26E-04 | 1,84E-02 |
| hsa-miR-22-3p | -1,11 | 9,94E-04 | 2,12E-02 |
| hsa-miR-199b-5p | -1,03 | 3,97E-04 | 1,35E-02 |
| hsa-miR-320c | -1,00 | 1,28E-04 | 7,42E-03 |
| hsa-miR-193a-3p | -0,99 | 3,21E-05 | 3,10E-03 |
| hsa-miR-199a-5p | -0,97 | 1,95E-03 | 3,02E-02 |
| hsa-miR-497-5p | -0,96 | 1,41E-03 | 2,48E-02 |
| hsa-miR-5703 | -0,93 | 7,10E-04 | 1,84E-02 |
| hsa-miR-152 | -0,92 | 4,36E-06 | 6,53E-04 |
| hsa-miR-30e-3p | -0,87 | 3,37E-06 | 5,69E-04 |
| hsa-miR-181b-5p | -0,86 | 4,27E-03 | 4,32E-02 |
| hsa-let-7c | -0,80 | 3,05E-03 | 3,75E-02 |
| hsa-miR-3663-3p | -0,77 | 6,19E-05 | 4,48E-03 |
| hsa-miR-211-3p | -0,76 | 2,80E-05 | 2,83E-03 |
| hsa-miR-31-5p | -0,73 | 2,79E-04 | 1,11E-02 |
| hsa-miR-126-5p | -0,72 | 1,87E-05 | 2,11E-03 |
| hsa-miR-185-5p | -0,69 | 1,85E-03 | 2,94E-02 |
| hsa-miR-423-5p | -0,64 | 1,92E-04 | 9,28E-03 |
| hsa-miR-3135b | -0,64 | 2,60E-03 | 3,42E-02 |
| hsa-miR-140-3p | -0,61 | 7,75E-04 | 1,85E-02 |
| hsa-miR-143-3p | -0,58 | 1,09E-03 | 2,25E-02 |
| hsa-miR-186-5p | -0,58 | 6,70E-05 | 4,68E-03 |
| dmr_3 | -0,57 | 7,34E-04 | 1,84E-02 |
| hsa-miR-1 | -0,55 | 2,13E-04 | 9,80E-03 |
| hsa-miR-203a | -0,54 | 8,79E-08 | 2,96E-05 |
| hsa-miR-6165 | -0,53 | 3,73E-03 | 4,08E-02 |
| hsa-miR-4306 | -0,50 | 2,06E-03 | 3,09E-02 |
| dmr_6 | -0,48 | 1,29E-03 | 2,47E-02 |
| hsa-miR-512-3p | -0,47 | 1,33E-04 | 7,50E-03 |
| hsa-miR-140-5p | -0,46 | 1,25E-03 | 2,43E-02 |
| hsa-miR-146a-5p | -0,45 | 1,50E-03 | 2,58E-02 |
| hsa-miR-133b | -0,43 | 4,37E-04 | 1,39E-02 |
| hsa-miR-3907 | -0,42 | 5,83E-06 | 7,88E-04 |
| hsa-let-7i-3p | -0,40 | 6,77E-08 | 2,75E-05 |
| hsa-miR-30c-2-3p | -0,39 | 1,69E-09 | 8,54E-07 |
| hsa-miR-4634 | -0,38 | 4,83E-03 | 4,47E-02 |
| hsa-miR-450a-5p | -0,34 | 1,02E-03 | 2,14E-02 |
| hsa-miR-4449 | -0,33 | 1,83E-04 | 9,28E-03 |
| hsa-miR-363-3p | -0,32 | 3,86E-03 | 4,11E-02 |
| hsa-miR-193a-5p | -0,30 | 1,76E-04 | 9,17E-03 |
| hsa-miR-874 | -0,28 | 3,40E-03 | 3,89E-02 |
| hsa-miR-145-3p | -0,27 | 1,75E-06 | 3,23E-04 |
| hsa-miR-7-2-3p | -0,26 | 1,02E-03 | 2,14E-02 |
| hsa-miR-455-5p | -0,24 | 2,01E-04 | 9,47E-03 |
| hsa-miR-202-3p | -0,22 | 4,34E-04 | 1,39E-02 |
| hsa-miR-139-5p | -0,20 | 2,20E-03 | 3,19E-02 |
| hsa-miR-143-5p | -0,19 | 3,63E-04 | 1,29E-02 |
| hsa-miR-499a-5p | -0,19 | 5,18E-04 | 1,50E-02 |
| hsa-miR-181a-2-3p | -0,17 | 4,28E-07 | 8,68E-05 |
| hsa-miR-139-3p | -0,16 | 4,16E-05 | 3,84E-03 |
| hsa-miR-214-5p | -0,14 | 3,17E-03 | 3,76E-02 |
| NC1_00000215 | -0,12 | 2,58E-03 | 3,42E-02 |
| hsa-miR-4633-5p | -0,12 | 4,66E-03 | 4,45E-02 |
| hsa-miR-190a | -0,11 | 1,43E-04 | 7,63E-03 |
| hsa-miR-30b-3p | -0,10 | 3,29E-03 | 3,82E-02 |
| NC2_00122731 | -0,09 | 7,30E-04 | 1,84E-02 |
| NC2_00106057 | -0,09 | 9,53E-04 | 2,10E-02 |
| hsa-miR-31-3p | -0,07 | 1,60E-03 | 2,66E-02 |
| NC1_00000197 | -0,07 | 8,96E-04 | 2,06E-02 |
| hsa-miR-29a-5p | -0,07 | 2,92E-04 | 1,14E-02 |
| hsa-miR-3149 | -0,06 | 4,62E-03 | 4,45E-02 |
| hsa-miR-100-3p | -0,06 | 3,18E-03 | 3,76E-02 |
| hsa-miR-3613-5p | -0,06 | 3,19E-03 | 3,76E-02 |
| hsa-miR-548b-5p | -0,06 | 1,29E-03 | 2,47E-02 |
| hsa-miR-548u | -0,06 | 1,41E-04 | 7,63E-03 |
| hsa-miR-30c-1-3p | -0,06 | 1,12E-03 | 2,27E-02 |
| NC2_00092197 | -0,06 | 3,15E-03 | 3,76E-02 |
| hsa-miR-1277-3p | -0,06 | 7,69E-04 | 1,85E-02 |
| hsa-miR-4775 | -0,06 | 5,64E-03 | 4,95E-02 |
| hsa-miR-374b-3p | -0,06 | 5,30E-03 | 4,78E-02 |
| hsa-miR-590-3p | -0,06 | 1,53E-03 | 2,60E-02 |
| hsa-miR-98-3p | -0,05 | 4,63E-03 | 4,45E-02 |
| hsa-miR-4803 | -0,05 | 2,40E-04 | 1,03E-02 |
| hsa-miR-548ai | -0,05 | 4,71E-03 | 4,47E-02 |
| hsa-miR-548ay-5p | -0,05 | 2,45E-03 | 3,42E-02 |
| hsa-miR-1252 | -0,05 | 1,19E-03 | 2,36E-02 |
| hsa-miR-3611 | -0,05 | 2,25E-03 | 3,23E-02 |
| hsa-miR-26a-1-3p | -0,05 | 6,00E-04 | 1,64E-02 |
| hsa-miR-4795-5p | -0,05 | 4,84E-04 | 1,49E-02 |
| hsa-let-7f-2-3p | -0,05 | 5,44E-04 | 1,53E-02 |
| hsa-miR-26a-2-3p | -0,05 | 5,56E-03 | 4,92E-02 |
| hsa-miR-3606-5p | -0,05 | 4,13E-03 | 4,23E-02 |
| hsa-miR-450b-5p | -0,05 | 2,02E-03 | 3,06E-02 |
| hsa-miR-302f | -0,04 | 3,99E-03 | 4,18E-02 |
| hsa-let-7a-3p | -0,04 | 2,52E-03 | 3,42E-02 |
| hsa-miR-5692b | -0,04 | 5,95E-05 | 4,47E-03 |
| hsa-miR-374c-3p | -0,04 | 5,65E-03 | 4,95E-02 |
| hsa-miR-3668 | -0,04 | 2,76E-03 | 3,52E-02 |
| hsa-miR-548c-3p | -0,04 | 4,63E-03 | 4,45E-02 |
| hsa-miR-302b-5p | -0,04 | 4,06E-03 | 4,18E-02 |
| hsa-miR-548ab | -0,04 | 4,38E-03 | 4,42E-02 |
| hsa-miR-586 | -0,04 | 2,39E-03 | 3,41E-02 |
| hsa-miR-6502-3p | -0,04 | 4,40E-04 | 1,39E-02 |
| hsa-miR-1245b-5p | -0,04 | 2,64E-03 | 3,43E-02 |
| hsa-miR-3669 | -0,04 | 1,99E-03 | 3,06E-02 |
| hsa-miR-19a-5p | -0,04 | 1,74E-03 | 2,80E-02 |
| hsa-miR-568 | -0,04 | 4,05E-04 | 1,35E-02 |
| hsa-miR-4752 | -0,04 | 3,93E-03 | 4,17E-02 |
| hsa-miR-4753-3p | -0,04 | 4,92E-03 | 4,53E-02 |
| hsa-miR-367-3p | -0,04 | 1,21E-03 | 2,38E-02 |
| hsa-miR-519c-3p | -0,04 | 1,36E-03 | 2,48E-02 |
| hsa-miR-488-3p | -0,04 | 3,11E-04 | 1,17E-02 |
| hsa-miR-190b | -0,04 | 4,04E-03 | 4,18E-02 |
| hsa-miR-3672 | -0,04 | 8,63E-04 | 2,03E-02 |
| hsa-miR-4798-5p | -0,04 | 9,12E-04 | 2,08E-02 |
| hsa-miR-122-3p | -0,04 | 3,07E-03 | 3,75E-02 |
| hsa-miR-302a-5p | -0,04 | 2,09E-03 | 3,11E-02 |
| hsa-miR-4699-5p | -0,04 | 3,79E-03 | 4,08E-02 |
| hsa-miR-4790-5p | -0,03 | 4,66E-03 | 4,45E-02 |
| hsa-miR-4762-3p | -0,03 | 2,61E-03 | 3,42E-02 |
| hsa-miR-4782-3p | -0,03 | 4,78E-03 | 4,47E-02 |
| hsa-miR-3134 | -0,03 | 3,65E-03 | 4,04E-02 |
| hsa-miR-518c-3p | -0,03 | 4,22E-03 | 4,30E-02 |
| hsa-miR-5580-5p | -0,03 | 2,51E-03 | 3,42E-02 |
| hsa-miR-609 | -0,03 | 2,76E-03 | 3,52E-02 |
| hsa-miR-559 | -0,03 | 1,80E-03 | 2,88E-02 |
| hsa-miR-4503 | -0,03 | 3,50E-03 | 3,98E-02 |
| hsa-miR-544b | -0,03 | 3,36E-04 | 1,21E-02 |
| hsa-miR-544a | -0,03 | 4,80E-03 | 4,47E-02 |
| dmr_316 | -0,03 | 7,28E-04 | 1,84E-02 |
| hsa-miR-1911-3p | -0,03 | 1,39E-03 | 2,48E-02 |
| hsa-miR-5007-3p | -0,03 | 4,05E-03 | 4,18E-02 |
| hsa-miR-302e | -0,03 | 2,58E-03 | 3,42E-02 |
| hsa-miR-223-5p | -0,03 | 3,60E-03 | 4,03E-02 |
| hsa-miR-3140-5p | -0,03 | 4,49E-03 | 4,45E-02 |
| hsa-miR-5590-3p | -0,03 | 4,66E-03 | 4,45E-02 |
| hsa-miR-548k | -0,03 | 2,42E-03 | 3,42E-02 |
| hsa-miR-3667-3p | -0,03 | 3,40E-03 | 3,89E-02 |
| hsa-miR-4766-3p | -0,03 | 1,33E-03 | 2,48E-02 |
| hsa-miR-4264 | -0,03 | 3,97E-03 | 4,18E-02 |
| hsa-miR-1256 | -0,03 | 2,98E-03 | 3,73E-02 |
| hsa-miR-3529-3p | -0,03 | 4,60E-03 | 4,45E-02 |
| hsa-miR-3973 | -0,03 | 8,83E-04 | 2,06E-02 |
| hsa-miR-3118 | -0,02 | 1,87E-03 | 2,94E-02 |
| hsa-miR-4711-3p | -0,02 | 4,82E-03 | 4,47E-02 |
| hsa-miR-1265 | -0,02 | 5,08E-03 | 4,64E-02 |
| hsa-miR-4747-5p | -0,02 | 5,30E-03 | 4,78E-02 |
| hsa-miR-4722-5p | 0,03 | 2,84E-03 | 3,59E-02 |
| hsa-miR-4714-3p | 0,03 | 4,62E-03 | 4,45E-02 |
| hsa-miR-4711-5p | 0,03 | 1,46E-03 | 2,53E-02 |
| hsa-miR-4303 | 0,03 | 3,70E-03 | 4,08E-02 |
| hsa-miR-659-5p | 0,04 | 2,76E-03 | 3,52E-02 |
| hsa-miR-1203 | 0,04 | 3,12E-03 | 3,76E-02 |
| hsa-miR-3177-3p | 0,05 | 3,57E-03 | 4,02E-02 |
| hsa-miR-3187-5p | 0,05 | 5,73E-03 | 4,96E-02 |
| hsa-miR-3177-5p | 0,05 | 3,82E-03 | 4,10E-02 |
| hsa-miR-1307-3p | 0,06 | 1,63E-03 | 2,67E-02 |
| hsa-miR-4304 | 0,06 | 5,79E-03 | 4,97E-02 |
| hsa-miR-5587-5p | 0,06 | 4,07E-04 | 1,35E-02 |
| hsa-miR-6718-5p | 0,06 | 3,07E-03 | 3,75E-02 |
| hsa-miR-34c-3p | 0,07 | 7,44E-05 | 5,03E-03 |
| hsa-miR-766-3p | 0,07 | 2,60E-03 | 3,42E-02 |
| hsa-miR-183-3p | 0,08 | 3,57E-03 | 4,02E-02 |
| hsa-miR-921 | 0,08 | 3,25E-03 | 3,81E-02 |
| hsa-miR-378f | 0,08 | 5,53E-03 | 4,92E-02 |
| hsa-miR-3130-5p | 0,08 | 2,10E-03 | 3,11E-02 |
| hsa-miR-5585-5p | 0,10 | 6,54E-04 | 1,77E-02 |
| hsa-miR-4494 | 0,10 | 1,33E-03 | 2,48E-02 |
| hsa-miR-4294 | 0,11 | 4,82E-03 | 4,47E-02 |
| hsa-miR-3935 | 0,12 | 2,55E-03 | 3,42E-02 |
| hsa-miR-4418 | 0,12 | 1,70E-03 | 2,76E-02 |
| hsa-miR-338-5p | 0,13 | 4,54E-03 | 4,45E-02 |
| hsa-miR-200a-5p | 0,13 | 5,51E-05 | 4,32E-03 |
| hsa-miR-3934-3p | 0,15 | 2,13E-03 | 3,12E-02 |
| hsa-miR-1208 | 0,16 | 3,28E-03 | 3,82E-02 |
| hsa-miR-1291 | 0,17 | 7,50E-04 | 1,85E-02 |
| hsa-miR-298 | 0,21 | 9,52E-04 | 2,10E-02 |
| hsa-miR-602 | 0,23 | 2,87E-03 | 3,62E-02 |
| hsa-miR-614 | 0,24 | 3,77E-03 | 4,08E-02 |
| hsa-miR-99b-3p | 0,26 | 2,61E-03 | 3,42E-02 |
| hsa-miR-4673 | 0,27 | 3,63E-03 | 4,04E-02 |
| hsa-miR-617 | 0,37 | 2,58E-03 | 3,42E-02 |
| hsa-miR-6075 | 0,37 | 2,20E-03 | 3,19E-02 |
| hsa-miR-4488 | 0,37 | 1,94E-03 | 3,02E-02 |
| hsa-miR-371a-5p | 0,39 | 2,56E-03 | 3,42E-02 |
| hsa-miR-5684 | 0,40 | 2,18E-04 | 9,80E-03 |
| hsa-miR-936 | 0,41 | 1,45E-03 | 2,53E-02 |
| hsa-miR-3197 | 0,43 | 2,59E-04 | 1,07E-02 |
| hsa-miR-378a-3p | 0,49 | 4,04E-03 | 4,18E-02 |
| hsa-miR-1233-1-5p | 0,49 | 1,36E-03 | 2,48E-02 |
| hsa-miR-182-5p | 0,50 | 5,59E-04 | 1,55E-02 |
| hsa-miR-4498 | 0,55 | 3,87E-04 | 1,35E-02 |
| hsa-miR-4733-5p | 0,55 | 2,25E-05 | 2,40E-03 |
| hsa-miR-4322 | 0,56 | 9,78E-04 | 2,11E-02 |
| hsa-miR-4746-3p | 0,56 | 3,75E-03 | 4,08E-02 |
| hsa-miR-1915-3p | 0,60 | 5,79E-03 | 4,97E-02 |
| hsa-miR-4538 | 0,61 | 3,02E-03 | 3,75E-02 |
| hsa-miR-4486 | 0,62 | 6,96E-04 | 1,84E-02 |
| hsa-miR-513b | 0,65 | 1,54E-03 | 2,60E-02 |
| hsa-miR-4632-5p | 0,66 | 1,39E-03 | 2,48E-02 |
| hsa-miR-1185-1-3p | 0,70 | 1,38E-03 | 2,48E-02 |
| hsa-miR-153 | 0,75 | 9,60E-04 | 2,10E-02 |
| hsa-miR-4695-5p | 0,83 | 2,01E-03 | 3,06E-02 |
| hsa-miR-1273e | 0,85 | 4,95E-04 | 1,50E-02 |
| hsa-miR-183-5p | 0,88 | 4,82E-04 | 1,49E-02 |
| hsa-miR-5001-5p | 0,90 | 5,19E-03 | 4,72E-02 |
| hsa-miR-762 | 0,91 | 5,01E-03 | 4,60E-02 |
| hsa-miR-4462 | 1,03 | 4,51E-06 | 6,53E-04 |
| hsa-miR-4505 | 1,05 | 5,17E-04 | 1,50E-02 |
| hsa-miR-1246 | 1,08 | 5,73E-03 | 4,96E-02 |
| hsa-miR-4530 | 1,10 | 4,80E-03 | 4,47E-02 |
| hsa-miR-513a-5p | 1,11 | 2,64E-04 | 1,07E-02 |
| hsa-miR-1224-5p | 1,12 | 1,12E-03 | 2,27E-02 |
| hsa-miR-197-5p | 1,18 | 5,66E-03 | 4,95E-02 |
| hsa-miR-4507 | 1,25 | 9,64E-04 | 2,10E-02 |
| hsa-miR-96-5p | 1,25 | 3,01E-04 | 1,15E-02 |
| hsa-miR-6125 | 1,29 | 2,26E-04 | 9,96E-03 |
| hsa-miR-4485 | 1,36 | 1,15E-03 | 2,30E-02 |
| hsa-miR-4672 | 1,37 | 1,92E-04 | 9,28E-03 |
| hsa-miR-4428 | 1,44 | 7,66E-04 | 1,85E-02 |
| hsa-miR-4299 | 1,63 | 1,36E-03 | 2,48E-02 |
| hsa-miR-1973 | 1,70 | 5,17E-04 | 1,50E-02 |
| hsa-miR-4497 | 1,79 | 1,23E-04 | 7,32E-03 |
| hsa-miR-4430 | 1,83 | 3,26E-04 | 1,20E-02 |
| hsa-miR-4653-3p | 1,85 | 8,67E-06 | 1,10E-03 |
| hsa-miR-3676-5p | 1,85 | 3,14E-03 | 3,76E-02 |
| hsa-miR-6132 | 1,95 | 9,49E-05 | 5,83E-03 |
| hsa-miR-4465 | 2,12 | 5,32E-04 | 1,52E-02 |
| hsa-miR-10a-5p | 2,16 | 1,63E-03 | 2,67E-02 |
| hsa-miR-375 | 4,50 | 1,02E-05 | 1,22E-03 |
